# Supplementary material for: Keeping it light: (re)analyzing community-wide datasets without major infrastructure
Source: Gigascience. 2018 Dec 13;8(2):giy159. doi: 10.1093/gigascience/giy159 (PMC6350038; doi:10.1093/gigascience/giy159)
Supplement: GIGA-D-18-00196_Revision-2.pdf [file giy159_giga-d-18-00196_revision-2.pdf]

GigaScience

Keeping it light: (Re)analyzing community-wide datasets without major infrastructure

--Manuscript Draft--

**Manuscript Number:**	GIGA-D-18-00196R2
**Full Title:**	Keeping it light: (Re)analyzing community-wide datasets without major infrastructure
**Article Type:**	Commentary
**Funding Information:**	Gordon and Betty Moore Foundation (GBMF4551) — Dr C. Titus Brown
**Abstract:**	DNA sequencing technology has revolutionized the field of biology, shifting biology from a data-limited to data-rich state. Central to the interpretation of sequencing data are the computational tools and approaches that convert raw data into biologically meaningful information. Both the tools and the generation of data are actively evolving, yet the practice of re-analysis of previously generated data with new tools is not commonplace. Re-analysis of existing data provides an affordable means of generating new information and will likely become more routine within biology, yet necessitates a new set of considerations for best practices and resource development. Here, we discuss several practices that we believe to be broadly applicable when re-analyzing data, especially when done by small research groups.
**Corresponding Author:**	Harriet Alexander, PhD<br><br>UNITED STATES
**Corresponding Author Secondary Information:**	
**Corresponding Author's Institution:**	
**Corresponding Author's Secondary Institution:**	
**First Author:**	Harriet Alexander, PhD
**First Author Secondary Information:**	
**Order of Authors:**	Harriet Alexander, PhD<br>Lisa K Johnson, BA<br>C. Titus Brown, PhD
**Order of Authors Secondary Information:**	
**Response to Reviewers:**	We have corrected the Johnson et al citation to be in press at GigaScience. We have also removed one citation (Kodama 2012) to conform to the 10 citation limit.
**Additional Information:**	
**Question**	**Response**
Are you submitting this manuscript to a special series or article collection?	No
**Experimental design and statistics**<br><br>Full details of the experimental design and statistical methods used should be given in the Methods section, as detailed in our Minimum Standards Reporting Checklist. Information essential to interpreting the data presented should be made available	No

Powered by Editorial Manager® and ProduXion Manager® from Aries Systems Corporation

in the figure legends.<br><br>Have you included all the information requested in your manuscript?	
If not, please give reasons for any omissions below.<br><br>as follow-up to "**Experimental design and statistics**<br><br>Full details of the experimental design and statistical methods used should be given in the Methods section, as detailed in our Minimum Standards Reporting Checklist. Information essential to interpreting the data presented should be made available in the figure legends.<br><br>Have you included all the information requested in your manuscript?<br><br>"	This is a
**Resources**<br><br>A description of all resources used, including antibodies, cell lines, animals and software tools, with enough information to allow them to be uniquely identified, should be included in the Methods section. Authors are strongly encouraged to cite Research Resource Identifiers (RRIDs) for antibodies, model organisms and tools, where possible.<br><br>Have you included the information requested as detailed in our Minimum Standards Reporting Checklist?	No
If not, please give reasons for any omissions below.<br><br>as follow-up to "**Resources**	This is a commentary piece to accompany the piece recently submitted by Johnson et al. As such, there are no data being analyzed.

A description of all resources used, including antibodies, cell lines, animals and software tools, with enough information to allow them to be uniquely identified, should be included in the Methods section. Authors are strongly encouraged to cite Research Resource Identifiers (RRIDs) for antibodies, model organisms and tools, where possible.<br><br>Have you included the information requested as detailed in our Minimum Standards Reporting Checklist?<br><br>"	
**Availability of data and materials**<br><br>All datasets and code on which the conclusions of the paper rely must be either included in your submission or deposited in publicly available repositories (where available and ethically appropriate), referencing such data using a unique identifier in the references and in the "Availability of Data and Materials" section of your manuscript.<br><br>Have you have met the above requirement as detailed in our Minimum Standards Reporting Checklist?	Yes

Manuscript
Click here to view linked References
Click here to access/download;Manuscript;Alexander_etal_OpEd_Reanalay

GigaScience, 2018, 1–3

doi: xx.xxxx/xxxx
Manuscript in Preparation
Commentary

COMMENTARY

Keeping it light: (Re)analyzing community-wide datasets without major infrastructure

Harriet Alexander[1,2], Lisa K. Johnson[1,3] and C. Titus Brown[1,3,4*]

[1]Population Health and Reproduction, University of California, Davis, CA, USA and [2]Biology Department, Woods Hole Oceanographic Institution, Woods Hole, MA, USA and [3]Molecular, Cellular, and Integrative Physiology Graduate Group, University of California, Davis, CA, USA and [4]Genome Center, University of California Davis, CA, USA

*ctbrown@ucdavis.edu

Abstract

DNA sequencing technology has revolutionized the field of biology, shifting biology from a data-limited to data-rich state. Central to the interpretation of sequencing data are the computational tools and approaches that convert raw data into biologically meaningful information. Both the tools and the generation of data are actively evolving, yet the practice of re-analysis of previously generated data with new tools is not commonplace. Re-analysis of existing data provides an affordable means of generating new information and will likely become more routine within biology, yet necessitates a new set of considerations for best practices and resource development. Here, we discuss several practices that we believe to be broadly applicable when re-analyzing data, especially when done by small research groups.

Key words: reproducibility; data reuse; open data;

Background

Advances in high-throughput, next-generation sequencing technologies have catapulted biology into a new computational era. In fields of biology that leverage sequencing data, the primary limiting step in the earlier stages of biological inquiry has increasingly shifted away from data generation to data analysis. Concomitant with the increasing emphasis on the computational processing of these data is the advancement of the computational tools available for such analyses: new computational approaches for the analysis of these data are constantly being created, tested, and proved worthy of use. Yet, outside of computational lab groups, the practice of re-analysis of previously generated data with new tools and approaches is not commonplace. Such re-analysis has great utility and will become more routine within the life sciences, yet re-analysis necessitates a new set of considerations for best practices and resource development.

Our interest in the issues surrounding re-analysis was spurred by a large-scale sequencing project: the Marine Microbial Transcriptome Sequencing Project (MMETSP), which generated 678 transcriptomes, spanning 396 different strains of eukaryotic microbial eukaryotes isolated from marine settings [1]. This dataset is an invaluable resource within the oceanographic community [2, 1], as it exponentially expands the accessible genetic information base of marine protistan life. Moreover, the MMETSP has created a uniquely useful test dataset for computational biologists. The MMETSP dataset spans a large evolutionary history of organisms, and all of the 678 transcriptomes were prepared and sequenced in a consistent way [2]. The sequencing project, which was completed in 2014, was originally assembled by the National Center for Genome Resources using a custom pipeline that employed the best available computational tools at the time [3, 4].

Since the original MMETSP analysis, new tools and techniques for the assembly of *de novo* transcriptomes from RNAseq data have been described and preexisting tools have been improved upon [5]. Moreover, new annotation tools and databases have become available. The transcriptome assembly project described in Johnson et al. [6] was designed to create

Compiled on: November 14, 2018.
Draft manuscript prepared by the author.

a streamlined and reproducible assembly framework that not only enables the re-analysis of these datasets, but creates a framework to facilitate easy and rapid re-analyses in the future.

These secondary data products of sequencing, such as annotated assemblies, should be viewed as hypotheses generated from the underlying biology, rather than some immutable "truth". As such these secondary data products can continue to be improved as new tools are developed. For example, we note that MacManes [7] described several limitations and challenges of current assembly technology and developed an improved Oyster River Protocol, which we could use to generate another, perhaps improved, MMETSP assembly.

Ultimately, such iterations on the original raw data have the potential to improve upon the secondary data products – the assembled transcriptomes and associated annotations that are relied upon by the broader community for biological inquiry. Through this process, we developed several practices that we believe to be broadly applicable when re-analyzing data, especially when done by small research groups.

Main text

Storage of secondary data products

Funding agencies and academic journals now mandate the deposition of raw data into digital repositories (e.g. NCBI Sequence Read Archive (SRA) and Gene Expression Omnibus, European Nucleotide Archive). Thus, to date, the majority of the sequence data that has been generated and published is openly available online for reference and use in other studies. The sharing and availability of raw data from high-throughput sequencing studies has been largely managed through the development of archival services such as the SRA, which was established as part of the International Nucleotide Sequence Database Collaboration (INSDC)[8]. The SRA currently contains more than 1.8e16 bases of information (~7e15 are open access)[1]. While a tremendous resource for biological inquiry, a major problem remains in that raw sequencing data is not the most directly useful form of sequencing data. Rather, biologists rely heavily upon the computationally generated secondary products of sequencing reads (e.g. assembled transcriptomes or genomes, annotations, associated count-based data, etc.). There is a dearth of these secondary products in central, publicly accessible databases, such as the Transcriptome Shotgun Assembly (TSA) Sequence Database.

In fact, a substantial proportion of these data products might be aptly categorized as "dark data," as they are largely undiscoverable and often archived independently in association with a publication or on private servers. Even more limiting, however, is that the guidelines for public databases such as the TSA specifically state that "Assemblies from sequences not directly sequenced by the submitter" should not be uploaded to the TSA, thereby excluding the potential for reassembled datasets to be made available and directly linked to preexisting BioProjects, BioSamples, TSAs, and SRA entries (https://www.ncbi.nlm.nih.gov/genbank/tsa/).

From the perspective of our MMETSP re-analysis, we argue the community needs more than a place to put the primary and secondary data products associated with a single publication. Ideally, the results of each re-analysis would be deposited in a discoverable location, but would have a coherent archival procedure that is lab-independent, easily searchable, and "forward discoverable" (i.e. when a new version of a data product is released, old versions can point to the new version). Moreover, such an archival platform would ideally document the full provenance of the secondary data product. Movement towards this kind of data archival system are being made both with the development of alternative scientific data publication models (e.g. the Research Object[9]) as well as integration of metadata models (such as the Resource Description Framework) onto existing scientific databases like the European Bioinformatics Institute (EBI) [10], but policies surrounding secondary data products will need to change.

Directly linking secondary data products to provenance of work-flow

In the absence of a community database specifically for the type of secondary product that was produced in this analysis, we opted to upload the assemblies, annotations, and counts to Zenodo (https://zenodo.org), a scientific data repository founded by CERN, which provided a DOI for the assemblies (https://doi.org/10.5281/zenodo.740440). The header information for each assembly was modified to contain the DOI. We then created a Github repository containing the scripts used to generate the assemblies. The repository was then archived with Zenodo, which generated a single DOI for the project (https://doi.org/10.5281/zenodo.594854).[2]

As such, the scripts used in the generation of transcriptomes are directly linked through a unique DOI to the data products that are listed in the directory. Since the scripts are easily accessible, they can be tweaked to re-analyze the primary sequence data using different parameters or tools, and the new pipeline and output files can be archived again with Zenodo using the same approach as above. Moreover, the Zenodo archival system will then automatically indicate the presence of other versions of a given repository such that a user might be sure to use the newest version of an assembly. In the future, such an approach might be further complemented by the integration of a JSON Linked Data file detailing the metadata for the assembly product, such as the pipeline used and previous versions of the assemblies.[3]

Conclusion

The Github-Zenodo framework presented here represents an efficent way for small research groups (i.e. a graduate student) to host and link both the code and results from large-scale re-analysis projects in a publicly accessible way. The direct linking of protocols and metadata to output data products is paramount in the data heavy future of scientific advancement. We also identified several lingering issues surrounding large scale re-analysis.

Actual computation on these large datasets is a non-trivial issue, as it requires access to facilities with sufficiently large, high-memory machines. Amazon Web Service instances and

1 As of 17 May 2018.

2 Individual components of the project are assigned specific DOIs, for example: translated peptide files: https://doi.org/10.5281/zenodo.745633; gff3 annotation files: https://doi.org/10.5281/zenodo.744702; annotation tables: https://doi.org/10.5281/zenodo.775129; quantification files: https://doi.org/10.5281/zenodo.746294.

3 It should be noted that uploading the assemblies to Zenodo was not an automated process. New versions of files on Zenodo must be manually curated. Since the start of this project, the Open Science Framework (OSF) and the accompanying automated command-line client, osfclient has been established. In the future, large-scale projects such as the assemblies created in this analysis may benefit from the integration of OSF command-line client by automatically uploading data products to an OSF project, which generate an OSF-specific DOI.

other "cloud" platforms, including XSEDE, provide flexible computing options, and are broadly accessible. Cloud-based systems, however, tend to be more expensive per computation hour than local resources. High Performance Computing (HPC) resources at local institutions represent another potential site of compute ability. However, HPCs can be temperamental and potentially balk at larger, more node-consuming procedures; moreover, bioinformatics tools may be poorly optimized for HPCs: Trinity, used in our pipeline, creates many small files for each run, and this repeatedly caused disk slowdowns on our HPC. The re-analysis by Johnson et al. [6] attempted to use both but ultimately found that the HPC provided the most consistent scalable automation for running hundreds of jobs in a cost efficient manner. However, more generally, we see no global solution for identifying and optimizing the global scientific cyberinfrastructure requirements for projects which require significant scaling; such considerations must be made on a project-by-project basis given the resources available to each lab.

Beyond the optimization of computational resources, we feel that there is a significant opportunity for scientific advancement with high-throughput sequencing projects in making data products "forward discoverable", because this makes it possible to improve downstream work without significant upstream investment. In an ideal future, a researcher might be automatically notified when a dataset that she is actively working on is updated or changes. This presents many social and technical challenges that will need to be solved if we are to take full advantage of public datasets.

Declarations

Competing interests

The authors declare that they have no competing interests.

Funding

Funding was provided from the Gordon and Betty Moore Foundation under award number GBMF4551 to C.T.B.

Author contribution

Conceptualized by H.A., L.K.J., and C.T.B. Written by H.A. and C.T.B. Edited and revised by H.A., C.T.B., and L.K.J. All authors read and approved the final manuscript.

References

1. Caron DA, Alexander H, Allen AE, Archibald JM, Armbrust EV, Bachy C, et al. Probing the evolution, ecology and physiology of marine protists using transcriptomics. Nature Reviews Microbiology 2016 nov;15(1):6–20. http://www.nature.com/doifinder/10.1038/nrmicro.2016.160.
2. Keeling PJ, Burki F, Wilcox HM, Allam B, Allen EE, Amaral-Zettler LA, et al. The Marine Microbial Eukaryote Transcriptome Sequencing Project (MMETSP): Illuminating the functional diversity of eukaryotic life in the oceans through transcriptome sequencing. PLoS Biology 2014 jun;12(6):e1001889. http://www.pubmedcentral.nih.gov/articlerender.fcgi?artid=4068987.
3. Simpson JT, Wong K, Jackman SD, Schein JE, Jones SJM, Birol I. ABySS: A parallel assembler for short read sequence data. Genome Research 2009 jun;19(6):1117–1123. http://www.ncbi.nlm.nih.gov/pubmed/19251739.
4. Huang X, Madan A. CAP3: A DNA sequence assembly program. Genome research 1999 sep;9(9):868–787. http://www.ncbi.nlm.nih.gov/pubmed/10508846.
5. Grabherr MG, Haas BJ, Yassour M, Levin JZ, Thompson DA, Amit I, et al. Full-length transcriptome assembly from RNA-Seq data without a reference genome. Nature biotechnology 2011 may;29(7):644–52. http://www.ncbi.nlm.nih.gov/pubmed/21572440.
6. Johnson LK, Alexander H, Brown CT. Re-assembly, quality evaluation, and annotation of 678 microbial eukaryotic reference transcriptomes. GigaScience in press;.
7. MacManes MD. The Oyster River Protocol: a multi-assembler and kmer approach for de novo transcriptome assembly. PeerJ 2018 aug;6:e5428. https://peerj.com/articles/5428.
8. Shumway M, Cochrane G, Sugawara H. Archiving next generation sequencing data. Nucleic Acids Research 2009 jan;38:D870–D871. http://www.ncbi.nlm.nih.gov/pubmed/19965774.
9. Bechhofer S, Buchan I, De Roure D, Missier P, Ainsworth J, Bhagat J, et al. Why linked data is not enough for scientists. Future Generation Computer Systems 2013;29(2):599–611.
10. Callahan A, Cruz-Toledo J, Ansell P, Dumontier M. Bio2RDF Release 2: Improved Coverage, Interoperability and Provenance of Life Science Linked Data. In: The Semantic Web: Semantics and Big Data Springer, Berlin, Heidelberg; 2013.p. 200–212. http://link.springer.com/10.1007/978-3-642-38288-8{_}14.
