# Supplementary material for: Keeping it light: (re)analyzing community-wide datasets without major infrastructure
Source: Gigascience. 2018 Dec 13;8(2):giy159. doi: 10.1093/gigascience/giy159 (PMC6350038; doi:10.1093/gigascience/giy159)
Supplement: Response-to-Reviewer-Comments_Original-Submission.pdf [file giy159_response-to-reviewer-comments_original-submission.pdf]

Author's Response To Reviewer Comments

Close

Response to Reviewers:
Keeping it light: (Re)analyzing community-wide datasets without major infrastructure by Alexander et al.

Reviewer #1: The commentary by Alexander et al. sheds light on several important gaps in the reproducibility in computational biology and discusses solutions but also practical issues based on the work of Johnson et al. I would like to raise a few minor points:

We thank the reviewer for the helpful feedback on our work. Please find how we have addressed the comments below (with our responses marked with *):

I am not sure if "push-button assembly framework" for a command line pipeline is a good expression. I could imagine that people without a strong computational background could misinterpret this. In my head I already see the PI going to his pet bioinformatician telling that the analysis can be done "just by pushing one button" and angrily asking why it always takes so long for her to analyze data.

* This is a fair point-- this framework is not "push-button" in the traditional, GUI sense of the word. (I can also imagine that particular PI-bioinformatician interaction occurring). We do believe that it is a streamlined and accessible approach, however. Therefore, we have changed the wording to "streamlined and reproducible assembly framework" (line 40).

I have a similar objections here: "The Github-Zenodo framework presented here represents a relatively low cost way for small research groups (i.e. a graduate student) to perform large-scale re-analysis projects in a publicly accessible way." I would rephrase this as GitHub and Zenodo are only for holding the code and the results. As the authors describe after that paragraph, the project required a vast amount of computational power to conduct the actual analysis and for this another type of infrastructure was needed. So "to perform large-scale re-analysis projects" the Github/Zenodo combo is not sufficient.

* This is a very good point. We agree that the frameworks utility lies mainly in the hosting-- not in the actual computational power required. We have changed the language to reflect that on line 139:

"The Github-Zenodo framework presented here represents an efficent way for small research groups (i.e. a graduate student) to host and link both the code and results from large-scale re-analysis projects in a publicly accessible way."

GitHub is sometimes misspelled (Github - "H" not capitalized).

* Thank you for catching the misspelling.

Figure 1 is not very expressive and I am not sure if it is really supporting the message.

* The figure was mainly designed to provide the idea of versioned pipelines being linked to versioned DOIs assigned to the outputs of the workflow (thus allowing the publication of multiple different datasets that are linked to alterations in the workflow). Given the feedback from both Reviewer 1 and Reviewer 2 we have decided to remove the figure.

Reviewer #2: I rather like this opinion piece. it makes a well reasoned argument about problems facing all of genomes, and more generally, all of “big data” science. I ahve just a few minor quips.

Thank you for your comments and thoughts, we have addressed your quips below:

I’m not sure about teh general utility of the MMETSp dataset as a testbed. This is because:

1a: The reads are too short. The vast majority are 50bp PE, which is not particularly representative of the read length most people are choosing for their de novo assembly projects, today, in 2018. How assemblers function with 50bp is likely different than how they function with 100bp.

* Yes, we agree with the reviewer that 50bp PE reads are far shorter than most assemblers will deal with now and in the future. As such, it is limited in that capacity. However, we do feel that the great diversity of life that is surveyed makes it an important datsets. The point about short reads (relative to the current norm) was raised in the review of the accompanying Johnson et al. paper and has been added in more detail in the discussion section there.

1b: The dataset is too big. 700 transcriptomes will challenge even the most computationally advanced labs. I do imagine a defined subset as being a good test-set. Maybe the authors could propose a subset that captures the taxonomic breadth and other dimensions of the dataset?

* This is a fantastic point. Yes, the datset is far too big to be a simple test dataset. We decided to address this point in the main text of the associated paper by Johnson et al. Taking the reviewer’s advice we have identified and listed 12 ‘High’ and 15 ‘Low’ performing assemblies that cover a cross-section of diversity from the MMETSP dataset. See Supplemental Figure 4 and in the text of Johnson et al.

I don’t see Fig1 referenced in the manuscript. In general, I’m not sure what Fig1 adds to it. Maybe knowing where it goes would help this?

* Thank you for catching this-- it looks as though there was an issue in the latex formatting on our end. However, given the similar response from Reviewer 1, we have decided to remove the figure.

Close
