# Supplementary material for: Keeping it light: (re)analyzing community-wide datasets without major infrastructure
Source: Gigascience. 2018 Dec 13;8(2):giy159. doi: 10.1093/gigascience/giy159 (PMC6350038; doi:10.1093/gigascience/giy159)
Supplement: Response-to-Reviewer-Comments_Revision-1.pdf [file giy159_response-to-reviewer-comments_revision-1.pdf]

Author's Response To Reviewer Comments

Close

We have corrected the Johnson et al citation to be in press at GigaScience. We have also removed one citation (Kodama 2012) to conform to the 10 citation limit.

Close
