# Supplementary material for: Keeping it light: (re)analyzing community-wide datasets without major infrastructure
Source: Gigascience. 2018 Dec 13;8(2):giy159. doi: 10.1093/gigascience/giy159 (PMC6350038; doi:10.1093/gigascience/giy159)
Supplement: Reviewer-1-Report-(Original-Submission) -- Konrad Foerstner — 2/7/2018 Reviewed [file giy159_reviewer-1-report-(original-submission).pdf]

**Reviewer Report**

**Title: Keeping it light: (Re)analyzing community-wide datasets without major infrastructure**

**Version: Original Submission** **Date:** 7/2/2018

**Reviewer name: Konrad Foerstner**

**Reviewer Comments to Author:**

The commentary by Alexander et al. sheds light on several important gaps in the reproducibility in computational biology and discusses solutions but also practical issues based on the work of Johnson et al.
I would like to raise a few minor points:

* I am not sure if "push-button assembly framework" for a command line pipeline is a good expression. I could imagine that people without a strong computational background could misinterpret this. In my head I already see the PI going to his pet bioinformatician telling that the analysis can be done "just by pushing one button" and angrily asking why it always takes so long for her to analyze data.
* I have a similar objections here: "The Github-Zenodo framework presented here represents a relatively low cost way for small research groups (i.e. a graduate student) to perform large-scale re-analysis projects in a publicly accessible way." I would rephrase this as GitHub and Zenodo are only for holding the code and the results. As the authors describe after that paragraph, the project required a vast amount of computational power to conduct the actual analysis and for this another type of infrastructure was needed. So "to perform large-scale re-analysis projects" the Github/Zenodo combo is not sufficient.
* GitHub is sometimes misspelled (Github - "H" not capitalized).
* Figure 1 is not very expressive and I am not sure if it is really supporting the message.

**Level of Interest**

Please indicate how interesting you found the manuscript: Choose an item.

**Quality of Written English**

Please indicate the quality of language in the manuscript: Choose an item.

**Declaration of Competing Interests**

Please complete a declaration of competing interests, considering the following questions:

- Have you in the past five years received reimbursements, fees, funding, or salary from an organisation that may in any way gain or lose financially from the publication of this manuscript, either now or in the future?

- Do you hold any stocks or shares in an organisation that may in any way gain or lose financially from the publication of this manuscript, either now or in the future?
- Do you hold or are you currently applying for any patents relating to the content of the manuscript?
- Have you received reimbursements, fees, funding, or salary from an organization that holds or has applied for patents relating to the content of the manuscript?
- Do you have any other financial competing interests?
- Do you have any non-financial competing interests in relation to this paper?

If you can answer no to all of the above, write 'I declare that I have no competing interests' below. If your reply is yes to any, please give details below.

I declare that I have no competing interests

I agree to the open peer review policy of the journal. I understand that my name will be included on my report to the authors and, if the manuscript is accepted for publication, my named report including any attachments I upload will be posted on the website along with the authors' responses. I agree for my report to be made available under an Open Access Creative Commons CC-BY license (http://creativecommons.org/licenses/by/4.0/). I understand that any comments which I do not wish to be included in my named report can be included as confidential comments to the editors, which will not be published.

I agree to the open peer review policy of the journal
