# Supplementary material for: Keeping it light: (re)analyzing community-wide datasets without major infrastructure
Source: Gigascience. 2018 Dec 13;8(2):giy159. doi: 10.1093/gigascience/giy159 (PMC6350038; doi:10.1093/gigascience/giy159)
Supplement: Reviewer-2-Report-(Original-Submission) -- Konrad Foerstner — 7/2/2018 Reviewed [file giy159_reviewer-2-report-(original-submission).pdf]

Reviewer Report

Title: Keeping it light: (Re)analyzing community-wide datasets without major infrastructure

Version: Original Submission **Date:** 7/2/2018

Reviewer name: Matthew Macmanes

Reviewer Comments to Author:

I rather like this opinion piece. it makes a well reasoned argument about problems facing all of genomes, and more generally, all of "big data" science. I ahve just a few minor quips.

1. I'm not sure about teh general utility of the MMETSp dataset as a testbed. This is because:
1a: The reads are too short. The vast majority are 50bp PE, which is not particularly representative of the read length most people are choosing for their de novo assembly projects, today, in 2018. How assemblers function with 50bp is likely different than how they function with 100bp.
1b: The dataset is too big. 700 transcriptomes will challenge even the most computationally advanced labs. I do imagine a defined subset as being a good test-set. Maybe the authors could propose a subset that captures the taxonomic breadth and other dimensions of the dataset?

2. I don't see Fig1 referenced in the manuscript. In general, I'm not sure what Fig1 adds to it. Maybe knowing where it goes would help this?

Level of Interest

Please indicate how interesting you found the manuscript: Choose an item.

Quality of Written English

Please indicate the quality of language in the manuscript: Choose an item.

Declaration of Competing Interests

Please complete a declaration of competing interests, considering the following questions:

- Have you in the past five years received reimbursements, fees, funding, or salary from an organisation that may in any way gain or lose financially from the publication of this manuscript, either now or in the future?
- Do you hold any stocks or shares in an organisation that may in any way gain or lose financially from the publication of this manuscript, either now or in the future?
- Do you hold or are you currently applying for any patents relating to the content of the manuscript?

- Have you received reimbursements, fees, funding, or salary from an organization that holds or has applied for patents relating to the content of the manuscript?
- Do you have any other financial competing interests?
- Do you have any non-financial competing interests in relation to this paper?

If you can answer no to all of the above, write 'I declare that I have no competing interests' below. If your reply is yes to any, please give details below.

I declare that I have no competing interests'

I agree to the open peer review policy of the journal. I understand that my name will be included on my report to the authors and, if the manuscript is accepted for publication, my named report including any attachments I upload will be posted on the website along with the authors' responses. I agree for my report to be made available under an Open Access Creative Commons CC-BY license (http://creativecommons.org/licenses/by/4.0/). I understand that any comments which I do not wish to be included in my named report can be included as confidential comments to the editors, which will not be published.

I agree to the open peer review policy of the journal
